# Incidence and Risk Factors of Infections Among Diffuse Large B-cell Lymphoma and Classical Hodgkin’s Lymphoma Patients in a Tertiary Care Center in Saudi Arabia: A Retrospective Cohort Study

**DOI:** 10.7759/cureus.35922

**Published:** 2023-03-09

**Authors:** Rakan H Alelyani, Ali H Alghamdi, Thamer A Almughamisi, Abdulrahman M Alshareef, Abdulaziz N Kadasa, Amir M Alrajhi, Abdullah K Alburayk, Ahmed S Barefah, Osman O Radhwi, Abdullah T Almohammadi, Salem M Bahashawan, Hatem M AlAhwal

**Affiliations:** 1 College of Medicine, King Abdulaziz University Faculty of Medicine, Jeddah, SAU; 2 Hematology, King Abdulaziz University Faculty of Medicine, Jeddah, SAU; 3 Hematology, King Fahd Medical Research Center, Jeddah, SAU

**Keywords:** rchop, diffuse large b-cell lymphoma, hodgkin's lymphoma, rituximab, infection, infection rates, rituximab therapy, hodgkin lymphona, rchop therapy

## Abstract

Introduction: Non-Hodgkin's lymphoma (NHL) ranked fourth among all cancer types in Saudi Arabia, as reported by the Saudi Health Council in 2015. Diffuse large B-cell lymphoma (DLBCL) is the most common histological type of NHL. On the other hand, classical Hodgkin's lymphoma (cHL) ranked sixth and had a modest tendency to affect young men more frequently. Over recent decades, DLBCL patients were treated with cyclophosphamide, doxorubicin hydrochloride, oncovin, and prednisolone (CHOP) alone. Adding rituximab (R) to the standard regimen (CHOP) shows significant improvement in overall survival. However, it also has a considerable effect on the immune system, impacting complement-mediated and antibody-dependent cellular cytotoxicity and causing an immunosuppressive state through modulating T-cell immunity via neutropenia, which can let the infection spread.

Aims and objectives: This study aims to evaluate the incidence and risk factors associated with infections in DLBCL patients in comparison to patients with cHL treated with doxorubicin hydrochloride (Adriamycin), bleomycin sulfate, vinblastine sulfate, and dacarbazine (ABVD).

Materials and methods: This study is a retrospective case-control study that included 201 patients acquired between January 1st, 2010, and January 1st, 2020. Sixty-seven patients had a diagnosis of cHL and had received ABVD, and 134 had DLBCL and had received rituximab. Clinical data were obtained from the medical records.

Results: During the study period, we enrolled 201 patients, of whom 67 had cHL, and 134 had DLBCL. DLBCL patients had a higher serum lactate dehydrogenase upon diagnosis than cHL (p = 0.005). Both groups have similar response rates with complete remission/partial remission. Compared to cHL, patients with DLBCL were more likely to have advanced disease when they first presented (stage III/IV, DLBCL: 67.3 vs. cHL: 56.5; p = 0.005). DLBCL patients had an increased risk of infection as compared to cHL patients (DLBCL: 32.1 % vs. 16.4%; p = 0.02). However, patients with a poor response to treatment had an increased risk of infection compared to patients with a favorable response regardless of the type of disease (odds ratio: 4.6; p = <0.001). When using multivariate analysis, it is revealed that unfavorable therapeutic response continues to be the only predictor raising the probability of infection in the population (odds ratio: 4.2; p = 0.003).

Conclusions: Our study explored all potential risk factors for the occurrence of infection in DLBCL patients who received R-CHOP versus cHL. The most reliable predictor of an increased risk of infection during the follow-up period was having an unfavorable response to medication. To assess these results, additional prospective research is required.

## Introduction

The fourth most common cancer in Saudi Arabia is non-Hodgkin's lymphoma (NHL), which accounted for 6.9% of all cancers reported by the Saudi Health Council in 2015 [1]. The most common histological subtype of NHL is diffuse large B-cell lymphoma (DLBCL), which accounts for nearly 50% of all other subtypes [1]. On the other hand, classical Hodgkin's lymphoma (cHL) ranked sixth among males and females, accounting for 3.6% of all cancers, with a slight predominance in males [1].

In the last two decades, monoclonal antibody therapy has significantly improved the prognosis of DLBCL [2,3]. Rituximab is a monoclonal antibody against CD20 used in DLBCL [4]. The standard treatment for DLBCL is cyclophosphamide, doxorubicin, oncovin, and prednisolone - rituximab (R-CHOP). Adding rituximab to CHOP (cyclophosphamide, doxorubicin hydrochloride, oncovin, and prednisolone) has improved progression-free survival and overall survival in DLBCL patients [4,5]. However, it has been reported that rituximab could significantly impact the immune system, affecting complement-mediated and antibody-dependent cellular cytotoxicity and leading to an immunosuppressive state by influencing T-cell immunity through neutropenia, which can allow infection to invade [5,6]. Infection is a significant source of morbidity and mortality, with neutropenic fever affecting 10-20% of treated lymphoma patients [7,8]. The rate of complete remission after receiving the first-line treatment of R-CHOP for six cycles in patients diagnosed with DLBCL is ≥75% [9,10]. The risk of relapse in the five years after completing the chemotherapy is low compared to other malignancies, which can affect less than 18% of these patients [11]. These findings are supported by recent studies showing low mortality for DLBCL patients in remission for two years [11-13].

cHL, like DLBCL, has become one of the most curable cancers in recent years [14]. At our center, cHL is treated using a combination therapy with doxorubicin, bleomycin, vinblastine, and dacarbazine (ABVD) [15]. According to the American Cancer Society, the International Prognostic Index (IPI) can assess the survival rate of NHL depending on age, stage, extranodal involvement, performance state, and the level of lactate dehydrogenase [16]. In comparison, many factors influence cHL survival, including the stage of the disease, having B symptoms, the bulk of the disease, being over the age of 45 years, being male, having a WBC greater than 15,000, and having hemoglobin less than 10.5 [17].

The risk of infections in DLBCL patients treated with rituximab has been reported in previous studies [18]. However, our study aims to assess the incidence and risk factors associated with infections in DLBCL patients treated with R-CHOP compared to patients with cHL treated with ABVD. We included all patients diagnosed with DLBCL treated with R-CHOP as the diseased group and cHL treated with ABVD as the control group from 2010 to 2020 in King Abdulaziz University Hospital.

## Materials and methods

Patients, eligibility criteria, and variables

Our retrospective case-control study included 201 patients from January 1, 2010, to January 1, 2020. A total of 134 patients were diagnosed with DLBCL and treated with R-CHOP, and 67 patients were diagnosed with cHL and treated with ABVD. All patients in this study were treated in the front-line setting. Patients' clinical information was obtained from the electronic hospital medical records. We included patients above the age of 18 years who were treated and followed up at King Abdulaziz University Hospital in Jeddah. We have excluded all pediatric patients (those under the age of 18 years), women who were pregnant, patients who were known to have HIV infection, and those with primary immunodeficiency diseases. Patients with a history of immunosuppressive drug exposure, as well as those who chose to continue their care in other facilities, were also excluded.

Data collection was performed based on the following variables: (1) demographics, including age at diagnosis, gender, weight, and body surface area (BSA). (2) Patient and disease-related information, including the date of diagnosis, stage at diagnosis, B symptoms (loss of weight, fever, night sweats), the bulk of the disease, extranodal sites, bone marrow involvement, and other comorbidities. (3) Relevant laboratory data before, during, and after chemotherapy, including serum creatinine, hemoglobin, white blood count (WBC), absolute neutrophil count (ANC), platelets (PLT), serum calcium, and lactate dehydrogenase (LDH). (4) Chemotherapy, including the type of protocol, date of starting and ending chemotherapy, number of cycles, and response to chemotherapy. (5) History and type of infection after starting treatment. Other variables were also collected, such as (6) the date of the last follow-up or death, (7) patient status (dead or alive), and (8) medication record. Infection events were identified when there was a positive culture, or clinical signs suggestive of an infectious process requiring antimicrobial therapy. For each infectious episode, the site of infection, the need for hospitalization, and the outcome were recorded. Body temperatures >38°C and the presence of symptoms or signs of inflammation at an anatomic site were considered clinically documented infections, regardless of whether cultures were positive. The patient's response to therapy was classified based on the International Working Group consensus response evaluation criteria in lymphoma (RECIL 2017).

Statistical analysis

When comparing the patient demographics, continuous data were reported as medians with ranges, and the Wilcoxon rank-sum test was used for comparison between the two groups. Categorical variables were compared using Fisher's exact test. Using logistic regression analysis, univariate analysis was completed to assess the effect of different risk factors on infection occurrence. A multivariate logistic regression model, including the underlying diagnosis, age, response to chemotherapy, stage at diagnosis, and history of diabetes, was included in the final model.

## Results

Patient's characteristics

We included 201 patients during the study period: 134 with DLBCL compared to 67 patients with cHL. The mean follow-up time for the DLBCL group was 24.7 months with a 95% confidence interval of 20.1-29.2 months, and for the cHL group, it was 31.4 months with a 95% confidence interval of 24.2-38.7 months. Patients with DLBCL were significantly older than cHL patients (mean age - DLBCL: 53.9 vs. cHL: 33.2, p < 0.001). Of note, patients with DLBCL were more likely to have diabetes mellitus (DM) (DLBCL: 28.4% vs. cHL: 6% p < 0.001) and hypertension (HTN) (DLBCL: 25.4% vs. cHL: 3% p < 0.001). There were no significant differences in gender, complete blood count (CBC) parameters, serum Ca, serum creatinine, chronic kidney disease (CKD), other malignancies, and the presence of neutropenia at diagnosis (Table 1). DLBCL patients had a higher serum LDH at diagnosis compared to cHL (Table 1). Both DLBCL and cHL patients had similar response rates with complete remission/partial remission rates of 80.2% and 87%, respectively. Patients with DLBCL were more likely to present with an advanced-stage disease compared to cHL (stage III/IV - DLBCL: 67.3 vs. cHL: 56.5, p = 0.005). The mortality rate over the study period among DLBCL patients (23.9%) was significantly higher compared to cHL patients (10.4%) with a p-value of 0.02. Patients with DLBCL were more likely to develop infections during the follow-up period than cHL patients (DLBCL: 32.1% vs. 16.4%, p = 0.02). There was no significant difference in neutropenia between both groups.

**Table 1:** Patients' characteristics and demographic DLBCL: diffuse large B-cell lymphoma; CBC: complete blood count; Dx: diagnosis; Hx: history; DM: diabetes mellitus; HTN: hypertension; CKD: chronic kidney disease; ANC: absolute neutrophil count; Hb: hemoglobin; PLT: platelets; LDH: lactate dehydrogenase; CR: complete response; PR: partial response; SD: stable disease; PD: progressive disease.

		DLBCL (frequency)	DLBCL (percentage)	Hodgkin’s lymphoma (frequency)	Hodgkin’s lymphoma (percentage)	P-value
Gender (n = 201)	Male	80	59.9	36	53.7	0.45
	Female	54	40.4	31	46.3	
		Mean	Range	Mean	Range	
Age (n = 201)		53.9	18-88	33.2	18-63	Less than 0.001
		Mean	Range	Mean	Range	
CBC at Dx (n = 201)	WBC	8.9	1.68-50.5	10.9	1.55-32.3	0.05
	ANC	7.1	0.37-87.8	11.7	0.6-79.1	0.02
	Hb	11.4	5.8-17.4	11.2	5.1-17.9	0.67
	PLT	318	45-910	394	11-856	0.72
	LDH	418.9	105-2926	236.1	126-862	0.005
	Serum creatinine	91.2	24-535	150.9	30->1000	0.34
	Ca	2.2	1.05-3.7	2.2	1.15-2.5	0.88
		Frequency	Percentage	Frequency	Percentage	
Hx of DM (n = 201)	Yes	38	28.4	4	6	Less than 0.001
	No	96	71.6	63	94	
									Frequency	Percentage	Frequency	Percentage	
Hx of HTN (n = 201)	Yes	34	25.4	2	3	Less than 0.001
	No	100	74.6	65	97	
		Frequency	Percentage	Frequency	Percentage	
Hx of CKD (n = 201)	Yes	3	2.2	0	0	0.55
	No	131	97.8	67	100	
		Frequency	Percentage	Frequency	Percentage	
Hx of other malignancies (n = 201)	Yes	2	1.5	0	0	0.55
	No	132	98.5	67	100	
		Frequency	Percentage	Frequency	Percentage	
Neutropenia at diagnosis (n = 189)	Yes	2	1.6	1	1.5	1.0
							No	121	98.4	65	98.5
												Response to therapy	CR/PR	97	80.2	47	87	0.39
	SD/PD	24	19.8	7	13													
			Frequency	Percentage	Frequency		Percentage	
Patient status	Alive	102	76.1	60	89.6	0.02
	Dead	32	23.9	7	10.4	
		Frequency	Percentage	Frequency	Percentage	
Hx of infection	Yes	43	32.1	11	16.4	0.02
	No	91	67.9	56	83.6	
		Frequency	Percentage	Frequency	Percentage	
Stage at diagnosis	1	14	13.1	2	4.3	0.005
	2	21	19.6	18	39.1	
	3	23	21.5	15	32.6	
	4	49	45.8	11	23.9	

Potential risk factors associated with the rate of infection

Using univariate analysis, we evaluated all possible factors affecting the prevalence of infections in the studied group (Table 2). We observed that cHL patients are less likely to develop an infection when compared to DLBCL patients (odds ratio: 0.4, p = 0.02). Patients with an unfavorable response to therapy had a higher risk of developing infectious complications compared to patients with a favorable response (odds ratio: 4.6, p < 0.001). The age, gender, and laboratory parameters at diagnosis, including neutropenia at diagnosis and stage at diagnosis, did not affect the risk of infection in the studied group.

**Table 2:** Univariate analysis assessing different factors affecting infection risk LDH: lactate dehydrogenase; DM: diabetes mellitus; HTN: hypertension; Neut: neutropenia; Dx: diagnosis; Tx: therapy.

	Odds ratio	P-value	95% CI	
Diagnosis	0.4	0.02	0.2	0.87
Age	1.02	0.03	1.0	1.03
Gender	1.53	0.18	0.82	2.87
LDH	1	0.3	0.99	1
Creatine	1	0.73	0.99	1
DM	0.5	0.07	0.2	1.05
HTN	0.5	0.76	0.24	1.07
Neut at Dx	0.18	0.16	0.02	2
Response to Tx	4.6	<0.001	2.1	10.5
Stage at Dx	1.3	0.16	0.91	1.8

Using multivariate analysis (Table 3), controlling for the underlying diagnosis, age, history of DM, history of HTN, and stage at diagnosis, an unfavorable response to therapy remains the single most important risk factor increasing the risk of infection in the studied population (odds ratio: 4.2, p = 0.003).

**Table 3:** Multivariate analysis assessing different factors affecting infection risk DM: diabetes mellitus.

	Odds ratio	P-value	95% CI	
Diagnosis	0.69	0.48	0.25	1.92
Age	1.0	0.97	0.97	1.03
DM	0.64	0.39	0.23	1.77
Response to therapy	4.2	0.003	1.62	10.88
Stage at diagnosis	1.0	0.99	0.67	1.5

## Discussion

Infectious complications and their outcomes among DLBCL patients who receive R-CHOP have not been well reported in our region. As rituximab has been shown to improve the response rate and the overall survival in DLBCL patients, further studies are required to assess its possible effect on infectious complications, especially in our geographical region. The occurrence of infection, febrile neutropenia (FN), leukocytopenia, and organ toxicity are possible adverse events of using chemotherapy to treat lymphomas. Rituximab's potential to interfere with humoral immunity may be what leads it to raise the risk of infection [19].

In this study, the infection rate in DLBCL was 32.1% and 16.4% in cHL (p = 0.02). A study conducted in Mexico showed similar results; among 265 DLBCL patients who received rituximab, 32.1% had infection with only 50% having known microbiology etiology [18]. Another study assessed the rate of infection and risk factors among 200 lymphoma patients who received chemotherapy and reported that 32.5% (27% NHL vs. 5.5% cHL) of all patients had an episode of infection [20]. They also reported an incident of FN, and it was 18.5% [20]. Similar to our results, a study that included 287 patients with DLBCL receiving R-CHOP showed the incidence of pneumonia was 29.3% during their chemotherapy [21]. The above-mentioned results are in keeping with the results reported in our study.

Considering patients' demographics and characteristics, DLBCL patients presented an older age than cHL patients, which is consistent with worldwide disease epidemiology [22]. DM and HTN were significantly higher in DLBCL compared to cHL. This can likely be explained by the older age of the DLBCL group. In this study group, being diagnosed with DLBCL or having an unfavorable response to treatment was considered a risk factor for developing infections. A study done by Wang et al. indicated all independent risk factors of developing infections in DLBCL during their immunochemotherapy, which included old age, known history of chronic renal disease, advanced stage, severe agranulocytosis, or pneumonia at the onset of their presentation [21].

Similar to our results, recent studies have reported that low hemoglobin levels will put patients at high risk of infection [23,24]. This was in contrast to our study, likely due to the relatively mild degree of anemia noted in our patient cohort, and thus, the effect of anemia would not have been well presented. As shown in our multivariate analysis, patients with disease progression were most likely to develop infections. Patients will be more susceptible to developing cytopenia and sequentially infections.

Limitations

Patients were enrolled from one single center; therefore, the results cannot be generalized to other populations. Due to the design of this study and the inclusion of all patients from 2010 to 2020, some initial patient-related data were missing; specifically, if there were any neutropenia at diagnosis, which 12 patients had; they did not have these data available at the time of diagnosis. Unfortunately, due to the retrospective nature of the study, results of cultures, imaging related to infections, and sites of infections were not available, which is a limitation of our study.

## Conclusions

Our study explored all possible risk factors for the occurrence of infection in DLBCL patients who received R-CHOP compared to cHL patients who received ABVD. Having an unfavorable response to therapy was the strongest indicator of the increased risk of infection during the follow-up period. Further prospective studies are needed to evaluate these findings.
